# Experimental and Field Assessment of Mineral–Cement–Emulsion Mixtures Containing Recycled Components

**DOI:** 10.3390/ma18214955

**Published:** 2025-10-30

**Authors:** Elżbieta Szafranko, Magdalena Czyż, Maciej Lis

**Affiliations:** Wydział Geoinżynierii, Uniwersytet Warmińsko-Mazurski, 10-719 Olsztyn, Poland

**Keywords:** mineral–cement–emulsion mixtures, recycling, sustainable development, road infrastructure

## Abstract

This study investigates the performance of mineral–cement–emulsion (MCE) mixtures produced with reclaimed asphalt pavement (RAP) and recycled mineral aggregates for use in road base layers. The aim was to evaluate the mechanical properties, field performance, and key factors influencing the cracking behavior of these sustainable cold-recycled mixtures. Approximately 160 laboratory tests were performed to determine indirect tensile strength (ITS), stiffness modulus (IT-CY), bulk density, and air-void content. The MCE mixture achieved an average ITS of 1.09 MPa and stiffness modulus of 5873 MPa after 28 days of curing, confirming compliance with design requirements. The field investigation of a test section showed good structural integrity and compaction, although several transverse cracks developed during the first year of service. The mechanistic interpretation attributed these cracks to combined cement hydration shrinkage and thermal contraction effects. The results indicate that MCE mixtures made with recycled materials can meet technical specifications while reducing environmental impact, provided that binder proportions and curing conditions are carefully optimized.

## 1. Introduction

The growing demand for sustainable and efficient building materials in recent years has encouraged both scientists and engineers to search for innovative solutions that could further engage the construction and civil engineering sector in the implementation of circular economy principles [1,2]. Mineral–cement–emulsion mixtures (MCE) are an example of technology that uses demolition materials while providing a complete product in terms of quality. Studies demonstrate that a mixture in which the main component is recycled material can meet all performance parameters. Such materials are increasingly more popular because they allow for the rational and ecological reuse of materials originating from dismantled bituminous surfaces, including ones that contain bituminous binders [3,4]. The development of cold recycling is well documented in numerous research papers from around the world [5,6,7,8,9,10,11,12,13,14,15,16], which proves its growing popularity. Furthermore, the introduction of requirements for MCE mixtures [17,18,19,20] and the publication of the Polish Catalogue of Typical Flexible and Semi-rigid Pavement Structures [19,20] by the road administration authority further highlight the importance and approval of this technology.

The purpose of this paper is to present an interdisciplinary analysis of the advantages and challenges, opportunities, and threats related to the development of this technology and the use of mixture, inclusive of technical, environmental, ecological, and social aspects. Having supplemented the information from the literature with a case study of an infrastructure development project in which MCE mixtures were used, the numerous advantages of the MCE mixtures were enumerated, although the sensitive points of the technology, which can constrain its applicability, were also determined. Additionally, the development of MCE technology opens up new possibilities for studies into the durability and environmental impact of the materials used. Further research can contribute to gaining a better understanding of the interactions between the mixture’s components, which is of key importance for the further development and optimization of this technology.

In recent years, cold in-place recycling (CIR) and cold recycled asphalt mixtures (CRMs) have attracted increasing attention as environmentally responsible alternatives to conventional hot-mix asphalt production. CIR techniques enable the in situ reuse of reclaimed asphalt pavement (RAP) by milling the existing pavement, adding stabilizing binders such as bitumen emulsion or foamed asphalt, and occasionally incorporating small quantities of cement or other hydraulic agents. Compared to traditional rehabilitation methods, CIR offers substantial reductions in energy consumption, CO_2_ emissions, and natural aggregate extraction, aligning closely with the principles of sustainable construction and a circular economy [21,22].

Jin et al. (2021) [23] performed an extensive laboratory and mechanistic–empirical (M–E) evaluation of CIR asphalt mixtures containing RAP, bitumen emulsion, and cement. Their results demonstrated that moisture conditioning using the Moisture-Induced Stress Tester (MIST) caused a 13–43% decrease in dynamic modulus and roughly a 20% reduction in low-temperature fracture energy. Despite this deterioration under moisture exposure, the M–E pavement analysis predicted satisfactory long-term performance under low-volume traffic, with reduced bottom-up cracking and improved smoothness (lower IRI values). These findings confirmed that although the material exhibits moderate stiffness and high moisture sensitivity, it can perform adequately when structural design and curing control are optimized. Importantly, they identified that an air-void content above ≈ 13% considerably increased the rate of stiffness loss and microcrack propagation, emphasizing the need for compaction control and moisture-resistant binder systems [23].

Complementary studies have provided deeper insights into the binder–aggregate interactions within cold recycled systems. Chen et al. (2020) [24] developed an improved design procedure incorporating curing-dependent strength growth, while Yang et al. (2019) [25] and Gan et al. (2022) [26] investigated the microstructural evolution of cement–asphalt composites. They found that early coalescence of asphalt droplets and rapid cement hydration can create localized stiffness gradients and shrinkage stresses at the asphalt–cement interface, which are critical in governing cracking resistance. Apeagyei and Diefenderfer (2013) [27] further validated, through field-cored specimens, that CIR and cold central–plant recycled layers can achieve mechanical performance comparable to hot-mix asphalt, provided that adequate curing, gradation, and binder dosage are ensured.

Collectively, these contemporary studies highlight that the durability and mechanical stability of cold recycled materials depend on the balance between hydraulic (cementitious) and bituminous phases, the control of moisture susceptibility, and the evolution of stiffness over time. Incorporating such findings into MCE mixture design supports a more mechanistic understanding of performance and informs the optimization of binder composition, curing regimes, and compaction practices. The present research builds upon these global developments by analyzing the field behavior of mineral–cement–emulsion (MCE) mixtures produced predominantly from recycled materials within Polish climatic and operational conditions.

### 1.1. Specificity of Mineral–Cement–Emulsion Mixtures

An MCE mixture is a conglomerate that can be used for making the base layer in paved road structures [23,24,25,26,27,28,29,30,31,32,33,34,35]. MCE mixtures contain reclaimed asphalt pavement aggregate to improve the mixture’s gradation, and bituminous emulsion and cement are used as binding agents [31,32,33,34,35,36,37,38,39,40,41,42,43,44,45].

The mixture’s composition, especially the presence of cement, can cause shrinkage and cracks in road layers. Cement and asphalt are commonly used in infrastructure building, including road surfaces, tunnels, and bridges. Cement-based materials offer benefits such as high compressive strength and corrosion resistance. However, they are rigid and have low deformability [16,17,18,19,20,21,22,23,24]. On the other hand, asphalt composites show excellent flexibility, outstanding viscoplastic properties, and good sound damping. However, they are prone to permanent deformation due to repeated loads, and have a huge carbon footprint [38,39,40,41,42]. One noteworthy example of an organic–inorganic composite is a mineral–cement–emulsion mixture. MCE mixtures possess diverse properties resulting from their composition, consisting mostly of reclaimed asphalt, cement, aggregates (sand), asphalt emulsion, and various additives [41,42,43,44,45,46,47,48]. Interactions in MCE mixtures include the effect of cement on the degradation process of asphalt emulsion and the effect of asphalt emulsion on cement hydration. Water released during the degradation of emulsion can be used for cement hydration. This water helps to solve the conflict between the hydrophobic nature of asphalt emulsions and the demand for water for hydration [45,46,47,48,49,50,51]. MCE mixtures are composed of reclaimed material or reclaimed material and mineral aggregate, cold-mixed with cement and asphalt emulsion, mixed in specific proportions, taking into consideration the amount of water. Cement in mixtures ensures the high strength of the material, while asphalt emulsion helps reduce shrinkage in the mixture by decreasing its stiffness [43,44,45]. These mixtures show a balanced distribution of aggregate sizes. Unlike cement- or asphalt-based materials, MCE mixtures combine the advantages of both of these types of material and also offset their disadvantages [35,36,37,38,39,40,41,42,43,44,45,46,47,48]. Considering the variety of applications and potential benefits, it is important to continue to work on improving the composition of MCE mixtures and their application techniques, which may further enhance the efficiency and durability of such mixtures. Ultimately, the properties of a mixture depend on the accurate choice of all ingredients, which often requires an in-depth analysis of each component material.

### 1.2. Advantages and Disadvantages of Using Mineral–Cement–Emulsion Mixtures

Recycling in road construction offers numerous benefits: it lowers the demand for mineral raw materials, decreases the costs of transporting aggregate, and substantially reduces or even eliminates the amount of waste from damaged (or old) road surfaces disposed of in landfills. Owing to the recycling of road surfaces, it is possible to reuse some materials, which having been properly reprocessed and mixed with binders such as asphalt and cement, in the right proportions, make up new materials with full value as a material [7,8,11].

Research completed by Chinese scholars [16,17] demonstrated that reclaimed asphalt combined with stabilized cement substrate can be used as recycled aggregate to make cement-stabilized mixtures using cold-recycling technology. Although an increase in the share of recycled materials led to some deterioration of their mechanical properties, the mixtures had sufficient durability and good performance.

An assessment of the environmental impact of disposal in landfills and use in road construction has been made in Denmark [18]. Ecotoxicity in water demonstrated a slight difference in the environmental impact of ash. Ash from electric furnaces, although not found to negatively affect the strength parameter of asphalt concrete, improves its stiffness. Using ash as a substitute for Portland cement in the production of concretes decreases their compressive strength, but increases their acoustic insulation, which may be useful when aiming to improve the thermal comfort, thereby contributing to reduced energy consumption in buildings.

The mechanical properties of mixtures, such as intermediate tensile strength or modules of strength, depend on both the type of waste materials used to make them and the way they are mixed and compacted.

Decision-makers and road authorities should take into account the economic and environmental consequences of the extraction of rock materials, the demand for which is in constant growth. Only by optimizing production processes and providing data on the materials made by producers is it possible to ensure an economic and ecological supply of aggregate materials and to eliminate legal, technical, and market-related barriers.

A higher share of reclaimed asphalt pavement (RAP) in a mixture deteriorates its strength, but has no significant effect on shrinkage, erosion, or capillary flow characteristics.

Based on the literature [7,8,10], the main benefits of using mineral–cement–emulsion mixtures made by incorporating recycled materials are as follows:

- Reduced consumption of natural resources without compromising the parameters of the mixture produced;
- Reduced costs of road development projects;
- A relatively simple production technology;
- ‘Cold-mixing’ technology means limiting the energy required to produce the mixture and reducing the carbon footprint.

In addition to generating economic and ecological benefits, MCE mixtures can contribute to improving road safety as they can provide better resistance to mechanical damage and reduce vulnerability to atmospheric influences, which is particularly important in regions with changeable weather. A mineral–cement–emulsion (MCE) mixture is an ecologically sustainable alternative to hot asphalt mixtures, and can bring significant economic benefits.

These are direct effects, but using recycled raw materials brings broader positive outcomes, such as less transport and lower amounts of contamination from the production of raw materials, which has a beneficial influence on the natural environment.

An MCE mixture is a conglomerate that can be used to build a base layer for roads. MCE mixtures contain some material recovered from old, degraded road surfaces; aggregate, which improves the grain size distribution; asphalt emulsions; and cement, which serves as a binder. The use of these components, especially cement, may be responsible for shrinkage and cracks in road layers.

Several authors of the research papers dealing with this subject [16,17,18,19,20] suggest the following weaknesses of MCE technology:

- Because such mixtures as a material present diverse and complex rheological, mechanical, and fatigue properties, they are placed between elastic and viscoelastic materials;
- The properties of the mixtures are strongly dependent on the quantity of binding agents they contain;
- Studies yield different results because of the content of heterogeneous materials such as reclaimed asphalt, e.g., from an old road surface; hence, the use of any materials from demolition requires careful selection and processing so as to ensure the cohesion and good quality of an MCE mixture;
- There is a general tendency to minimize the content of cement in MCE mixtures. Frequently, the standards set the maximum cement content at 1–2%, where a higher cement content would be acceptable. Creating an MCE mixture in which bituminous binding dominates hydraulic binding is still an unsolved problem.

### 1.3. Opportunities and Barriers to the Development of Technology Using Recycled Raw Materials

Cold recycling technologies with the use of mineral–cement–emulsion (MCE) mixtures have been gaining increasing interest in European, Asian, and North American countries. The growing demand for sustainable road infrastructure development, the implementation of a closed-cycle economy, and economic and environmental pressures are stimulating more intensive research on the use of recycled materials, including reclaimed asphalt pavement (RAP), in MCE mixtures. For the mentioned type of technology, both opportunities and barriers are rooted in the technology’s advantages and disadvantages, which are well described in the literature.

Opportunities for the development of MCE technology with recycling:

As demonstrated in some studies [6,24,44], the main advantages of MCE technology with the use of reclaimed materials from road demolition are its low cost, the recycling of local and reclaimed materials, and reduced CO2 emissions caused by heating and transporting the processed materials. The use of asphalt emulsion as a binder means that the final product can be made in ambient temperatures, which lowers energy consumption [7].

The recovered building materials used most often are reclaimed asphalt pavement (RAP), concrete aggregates, and, less often, fly ash and metallurgical slag, helping to limit the exploitation of natural aggregates and reduce the amount of waste deposited in landfills [47].

Experiments have also shown that MCE mixtures containing cement and emulsified asphalt can achieve comparable strength and durability parameters as classic pavement surfaces, including high crack resistance and dimensional stability [45]. By using cement with a hydraulic additives, it is possible to obtain better stiffness and durability at low temperatures.

Barriers to and limitations of using MCE mixtures made from reclaimed materials:

Despite the numerous benefits, the development of MCE technology with waste recycling also faces some significant limitations. The ones most often mentioned are as follows:

- Variability in the quality of recycled materials, especially of reclaimed asphalt pavement and concrete aggregates, which may contain contaminants (e.g., gypsum, wood, thermally cracked asphalt). Such raw materials need detailed laboratory analysis and sorting [48].
- Problems with the consistency of the mixture: the recycling of heterogeneous materials can result in the occurrence of weak points in the structural layers of a road [49,50]. There is a lack of consistent design guidelines in many countries, and there are no uniform technological standards or procedures for designing road surfaces with MCE mixtures. There are differences at every stage of the design process, including the selection of materials and the evaluation of the mixture produced [18,19,20].
- Possibilities for implementing MCE technology on smaller development projects carried out by local authorities can be limited by the lack of proper equipment or infrastructure as well as insufficient knowledge and experience, which can also be a barrier to construction companies lacking adequate resources.
- Lower frost resistance and higher water absorbability: mixtures containing reclaimed aggregate in their composition may be prone to higher water absorption and be structurally weaker to repeated freeze–thaw cycles [25].

It is also worth mentioning that the small number of large-scale projects with MCE mixtures prevents the extensive validation of this technology. Hence, the development of MCE mixtures with recycled materials requires more intensive research, the implementation of pilot test road sections, and adequate legislative and financial incentives.

MCE mixtures made with recycled materials have a future as a direction in the development of road construction technologies, in line with the assumptions of the circular economy and sustainable construction industry. Although their production faces technical, organizational, and formal barriers, there are numerous research papers and positive research results that confirm the potential of such mixtures as long-lasting, more ecofriendly, and economically feasible building materials. The key to the advancement of this technology will be to refine the design methods, develop the raw materials base, and educate the engineering community.

Another challenge is to provide adequate education and training programs for road engineers and technicians, which will help to better understand and use MCE technology in practice. This, in turn, can help to overcome the technical and operational barriers that currently suppress the wider application of this technology.

In order to verify the information gathered from source references, the case study described in this article was conducted.

## 2. Materials and Methods

This article presents the results of a study performed by the authors. Tests of the basic parameters of MCE mixtures, such as the content of free spaces, intermediate tensile strength, or the modulus of stiffness, allow for the determination of the necessary granulation with mineral material and to calculate the amount of necessary binders. The basic tests at the stage of designing the composition of an MCE mixture are sufficient to determine its basic parameters, but do not allow for a broader analysis of the way the tested material will behave, especially in the context of its future work in the construction of a road surface.

The primary purpose of this study was to evaluate the mechanical properties and in-service performance of mineral–cement–emulsion (MCE) mixtures manufactured predominantly from recycled materials, including reclaimed asphalt pavement (RAP) and recycled mineral aggregates. The study aimed to verify whether such mixtures can meet the structural and durability requirements for use in road base layers under Polish climatic conditions.

To achieve this, the following objectives were defined:

- To perform a comprehensive laboratory evaluation of MCE mixtures, determining indirect tensile strength (ITS), stiffness modulus (IT-CY), bulk density, and air-void content based on approximately 160 individual tests.
- To conduct field observations on a trial road section, assessing compaction quality, surface condition, and the occurrence of transverse cracking during the first year of service.
- To provide a mechanistic interpretation of the observed field behavior, identifying the main factors contributing to cracking despite compliance with technical specifications.
- To propose practical recommendations for optimizing binder composition and curing procedures to improve the long-term performance of MCE mixtures with recycled components.

These objectives form the foundation for the analytical and discussion sections that follow, linking laboratory performance to actual field behavior.

MCE surfaces on roads in the categories KR3-KR4 built under the contract for the construction of the S7 expressway were submitted for analysis. The recipes were analyzed together with the production technology process. In addition, the grain size, content of free spaces, bulk density, intermediate tensile strength, modulus of stiffness IT-CY, bearing capacity: dynamic modulus Evd after 7 days, bearing capacity: dynamic modulus Evd after 28 days, compaction index, and content of the binder were tested. Approximately 160 tests were performed for each parameter. The study demonstrated that all technical specification requirements were satisfied.

The experimental program was developed to evaluate the mechanical behavior and field performance of mineral–cement–emulsion (MCE) mixtures containing recycled aggregates. The study focused on sections of the trial road where visible transverse cracks had appeared. Based on a detailed surface analysis, it was determined that performing approximately 160 individual tests would provide a representative data set capturing the variability of material properties across both cracked and intact zones. This number therefore reflects the extent of the affected pavement area rather than a predetermined testing matrix.

The laboratory testing program included the determination of indirect tensile strength (ITS), stiffness modulus (IT-CY), bulk density, and air-void content. The following testing standards were applied:

− PN-EN 12697-23:2017-07 [52]—Determination of indirect tensile strength of bituminous mixtures;
− PN-EN 12697-26:2012, Annex C [53]—Stiffness modulus (IT-CY method);
− PN-EN 12697-6 [54]—Determination of bulk density;
− PN-EN 12697-8 [55]—Determination of air voids content.

These European standards, widely adopted in Polish road construction practice, ensured compatibility with national and international design guidelines. In addition, a field verification program was implemented, consisting of layer compaction measurements, surface condition assessments, and the systematic monitoring of transverse cracking during the first year of service.

The field observation methodology was developed by the authors to provide a consistent evaluation of the pavement’s in situ behavior. The results from both laboratory and field measurements were integrated to verify the relationship between compliance with technical specifications and actual long-term performance, as presented in Section 3 and discussed in Section 4.

### 2.1. Composition of the MCE Mixture Used

The mixture used to build the lower layer of the road’s base substructure in the aforementioned construction project had the basic composition as specified Table 1.

When designing the mixture, the grain size content of reclaimed asphalt pavement to MCE was determined (grain size distribution curves for the mineral cement mixture).

The MCE mixture with the properties given in Table 2 was designed.

### 2.2. MCE Layer Construction Technology

According to the project, the purpose of the construction work was to build an MCE substructure layer with a thickness of 20 cm. In accordance with the provisions of the contact, the Contractor prepared test sections in order to determine the following:

- Whether the equipment for laying and compacting the aggregate was adequate;
- The thickness of a loose layer of material necessary to obtain the set thickness of the compacted layer;
- The number of roller passes necessary to achieve the required compactness and bearing capacity;
- The usefulness of the proposed recipe for making the substructure layer with the MCE mixture;
- The usefulness of the equipment and the choice of means of transport to deliver the mixture;
- The homogeneity of the MCE mixture layer and the efficiency of the compaction equipment;
- The parameters of the layer made of the MCE mixture.

Prior to constructing a section of the road, the suitability of the materials and mixtures of materials for making an MCE mixture was confirmed by comparing these materials with the ones used for making the MCE mixture at the stage of recipe development.

A section of the improved lower subbase, built and accepted, served to lay on it a profiled and compacted 20 cm thick layer containing an MCE mixture. This layer was profiled with a paver and compacted with a smooth wheeled roller and pneumatic tired roller.

Once the MCE layer had been made as described above, the required values of the following parameters were obtained:

- After 7 days: E2 > 130 MPa (EVD ≥ 65 MPa);
- After 28 days: E2 > 180 MPa (EVD ≥ 90 MPa);
- Compaction index: Is ≥ 098.

The test section was then considered to have been completed.

The following tests were made during the construction work:

- Quality of the mineral mixture—visual assessment;
- Approximate content of grading materials;
- Dosing of binders (cement and asphalt emulsion);
- Homogeneity and surroundings—visual assessment;
- Thickness of the layer after compaction;
- Width and the transverse inclination of the layer;
- Wet particle size distribution (grading) of the mixture of aggregate with reclaimed asphalt, according to the standard PN-EN 933-1 [62], tested once daily;
- Moisture content of the mixture according to PN-EN 1097-5 [63], should correspond to ±20% of mopt tested once daily;
- Content of asphalt in the reclaimed asphalt emulsion based on extraction according to PN-EN 12697-1: tested once daily;
- Content of cement in the mixture, according to the manufacturer’s documents: constant control;
- Content of emulsion in the mixture: constant control;
- Properties of cement: in case of doubt;
- Properties of emulsions: in case of doubt;
- Properties of water: in case of a dubious source of water.

## 3. Results

Once the mixture had been incorporated into the road layer, the following tests were run:

- Void content—once (three samples) per 3000 m^2^;
- Intermediate tensile strength ITS after 28 days—once (three samples) per 3000 m^2^;
- Stiffness modulus IT-CY after 28 days—once (three samples) per 6000 m^2^;
- Bearing capacity of the subbase layer; deformation modulus E2 or dynamic modulus EVD after 7 days or after 28 days if possible, twice per layer;
- Thickness of the layers: three points on a daily plot (min. 1/2000 m^2^);
- Compaction index: once per 3000 m^2^;
- Checking geometric features.

On the basis of the constructed test section and the tests performed, the following conclusions were drawn:

- Positive quality parameters of the layer were obtained—meeting the ST requirements with respect to compaction and load-bearing capacity;
- Positive intermediate tensile strength after 28 days was obtained;
- Positive resistance of the samples to water was obtained;
- The proper stiffness modulus IT-CY was obtained;
- On the basis of the evenness control results, it was determined that the building equipment for spreading and compacting the mixture was suitable and ensured the construction of a homogenous and smooth surface;
- The layer’s thickness control (after compaction) enabled determination of the minimum thickness (+4.0 cm relative to the designed thickness) needed to obtain the required thickness of 0.20 m of the finished layer after compaction;
- The layer’s compaction control enabled determination of the number of passes of each type of a roller ensuring the achievement of the required compaction index. The above number was determined as four passes with a heavy steel wheeled roller in order to compact the deep subbase and four passes with a pneumatic tired roller in order to seal the surface of the layer.

**Figure 1:**
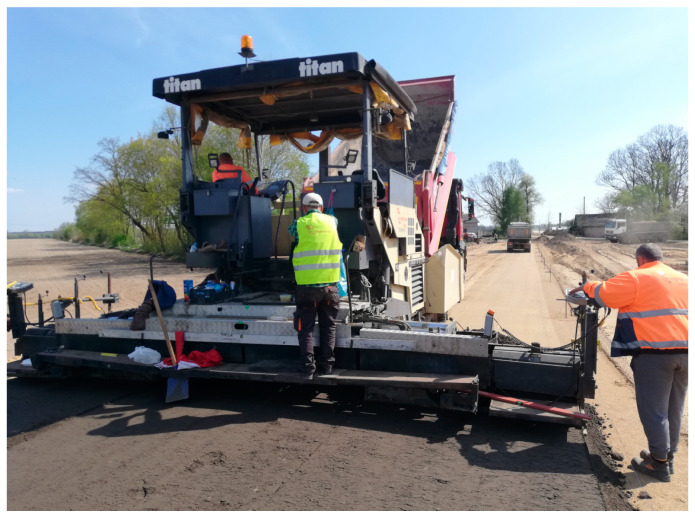
Spreading the MCE mixture (source: the author).

**Figure 2:**
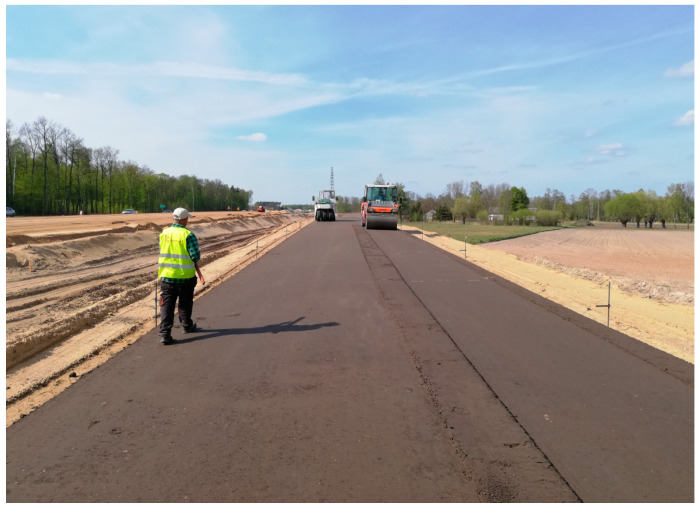
Compaction of the MCE mixture (source: the author).

The sections of the subbase built with the use of the MCE mixture underwent a complete set of tests for determination of particle size distribution, void content, bulk density, intermediate tensile strength, stiffness measured with the IT-CY method, dynamic modulus, Evd, compaction index, and content of binders. The process of paving is shown in Figure 1 and Figure 2.

**Figure 3:**
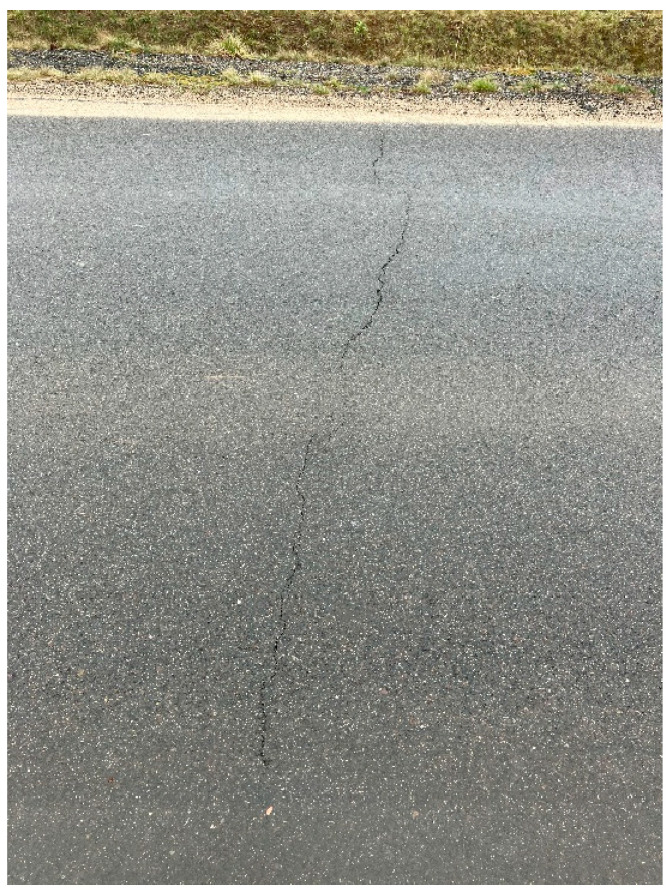
Cracks on the surface (source: the author).

**Figure 4:**
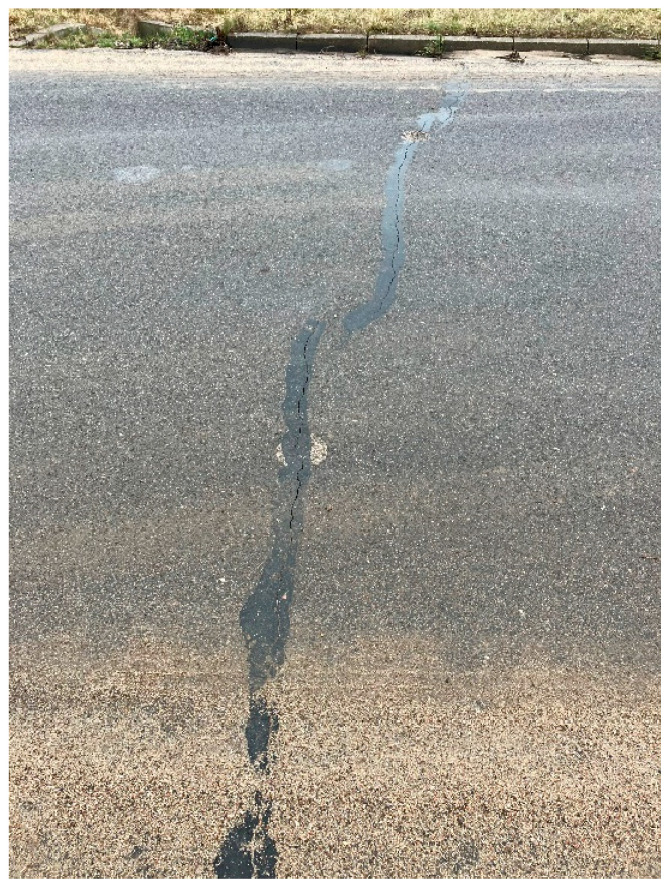
Repairing a crack (source: the author).

The reports summarizing the tests showed the compliance of the parameters of the tested samples with the design assumptions. However, the surfacing layer exhibited many transverse cracks in the first year of use and is now subject to constant repair. The condition after the first year of use is shown in Figure 3 and Figure 4.

Table 3 summarizes the main test results obtained from approximately 160 measurements conducted for each evaluated parameter. The data show that all mixtures met the specification limits; however, noticeable variation was recorded in stiffness and tensile strength, reflecting the heterogeneous nature of the recycled materials.

The obtained results confirm that the designed MCE mixture achieved appropriate stiffness and cohesion while maintaining acceptable compaction and void content. The variation in ITS and stiffness values illustrates the sensitivity of recycled mixtures to changes in binder distribution and aggregate gradation, which can explain the differences in field performance discussed later.

## 4. Discussion

The tests carried out on the construction site of a test road section provided information on both the parameters of the materials used and the construction technology. Positive values of the quality parameters of the test road layer were obtained, passing the ST requirements for both compaction and load-bearing capacity; in addition, a positive value of the intermediate tensile strength after 28 was determined. The subsequent tests confirmed the resistance of samples to water. The IT-CY stiffness modulus calculated was also positive. Based on the determination of the layer’s thickness control (after compaction), the minimum thickness (+4.0 cm more than the designed thickness) necessary to obtain the required thickness of 0.20 m of the finished layer after compaction was determined. The study positively verifies the data presented in the relevant source references.

Based on the surface evenness test, it was determined that the building equipment for spreading and compacting the material was suitable and guaranteed the obtaining of a uniform, smooth surface. The number of passes of each type of roller, ensuring the required compaction index, was also determined. Four passes with a smooth wheeled roller were required for compaction of the deep part of the subbase and four passes with a pneumatic tired roller were required to seal the surface.

The test reports demonstrated the compliance of the tested samples with the assumptions made at the stage of designing the road layer. However, the finished road surface developed numerous transverse cracks as early as during the first year of use, and now requires continuous repairs. The data regarding the state of the surface after a year of use implicates the need to continue research and to be aware of possible problems related to the application of MCE mixtures made with recycled materials to build a road subbase.

Although all of the laboratory and field tests confirmed that the MCE layer satisfied the required technical specifications, its performance in service revealed several defects, primarily transverse cracking. This apparent contradiction between compliance and performance can be explained by the limitations of current specification-based testing. The existing standards focus on short-term strength and stiffness, but do not adequately capture the mixture’s time-dependent behavior or its response to thermal and shrinkage stresses.

In practice, MCE layers may become excessively stiff immediately after construction due to rapid cement hydration, which limits their ability to recover from stress induced by temperature fluctuations or traffic loading. Consequently, even though the mixture meets ITS and IT-CY modulus requirements, internal stresses accumulate and lead to cracking.

Therefore, to improve field performance, the assessment of MCE mixtures should not rely solely on standard mechanical indicators, but should also include parameters describing shrinkage strain, fatigue resistance, and stiffness evolution over time. This approach would help align technical compliance with real-world durability and reduce the risk of premature cracking.

This case study places emphasis on the monitoring and constant evaluation of physical and mechanical properties of an MCE mixture after its application. This approach enables the identification of areas that need to be improved and the adjustment of the mixture’s parameters to meet the specific requirements of a given development project. Previous Polish experiments concerning road construction prove that incorrectly defined requirements for a cold recycling mixture may result in the production of an excessively stiff mixture, which will lead to the appearance of transverse fractures on the finished road surface. Field observations, laboratory tests and analyses to identify the causes of surface cracks led to some modification of the earlier parameter values. The aim of this change was to create a trend toward designing a cold recycling mixture that would be more elastic. In laboratory conditions, MCE mixtures designed in line with the new requirements had lower stiffness, but it is only through further inspections of the road sections currently under construction that we will be able to verify whether the achieved decrease in the mixture’s stiffness is sufficient for eliminating the risk of reflection cracking.

Despite meeting all specification requirements regarding compaction, tensile strength, and stiffness modulus, transverse cracks were observed in the MCE layer during the first year of service. This phenomenon may be attributed to a combination of shrinkage and stiffness-related mechanisms resulting from the complex interaction between cement and asphalt emulsion in the composite binder system.

The main causes of cracking can be identified as follows:

(1) The hydration shrinkage of cement, which leads to internal tensile stresses during early curing stages;
(2) The thermal contraction of the rigid MCE layer during temperature drops, which is not fully compensated for by the viscoelastic relaxation of the asphalt emulsion;
(3) The stiffness mismatch between the MCE base and the flexible asphalt overlay, which promotes stress concentration at the interface.

Although the MCE mixture achieved satisfactory ITS and IT-CY modulus values, the relatively high cement content (3%) likely increased brittleness, reducing the material’s ability to accommodate tensile strain. Similar conclusions were reported by Miljković et al. (2019) and Gan et al. (2022), who observed that the increase in cement dosage in cement–asphalt composites enhances stiffness but reduces fracture resistance [10,26].

Recent microstructural studies (Yang et al., 2019 [25]; Pi et al., 2019 [39]) demonstrate that the degradation of asphalt emulsion during cement hydration produces local zones of weak adhesion and capillary porosity, which act as microcrack initiators. Furthermore, Kukiełka and Gałan (2023) [45] highlighted that inappropriate grain size distribution may amplify these effects by limiting the relaxation capacity of the matrix.

Future studies should, therefore, focus on quantifying the shrinkage and fatigue behavior of MCE mixtures with variable cement-to-emulsion ratios and optimized aggregate gradation. Incorporating shrinkage-reducing admixtures or polymer-modified emulsions could help mitigate cracking susceptibility while maintaining adequate stiffness and load-bearing capacity.

The interaction between cement and asphalt emulsion in MCE mixtures plays a crucial role in determining their mechanical and volumetric stability. During hydration, cement particles accelerate the breaking of asphalt emulsion, releasing water that simultaneously contributes to cement hydration and to the coalescence of bitumen droplets. This process leads to the formation of a dual-phase composite structure composed of hydrated cement paste and dispersed asphalt binder.

However, this synergistic mechanism also generates internal stresses caused by volumetric changes in both phases: cement hydration induces shrinkage, while the asphalt phase undergoes viscoelastic deformation. When the cement content is high or the hydration process proceeds too quickly, these stresses may accumulate and cause early microcracking.

Binder optimization therefore focuses on achieving a balanced stiffness between the hydraulic and bituminous phases. Reducing the cement content to the lowest level to ensure adequate strength, using polymer-modified emulsions, and incorporating elastic or pozzolanic additives (e.g., rubber powder, fly ash, or slag) can significantly improve the crack resistance and long-term flexibility of MCE layers.

These strategies support the development of more durable and environmentally sustainable MCE mixtures, aligning with the objectives of a circular economy and performance-based pavement design.

To summarize, an interdisciplinary analysis of MCE technology underlines its potential as well as the complexity of problems related to its practical application. The development of norms and standards that will be better suited to the reality of road construction and use could be of key importance to take full advantage of the benefits offered by MCE mixtures.

## 5. Conclusions

This case study emphasizes the importance of monitoring and constant assessment of the physical and mechanical properties of a mixture after its application. This approach enables the identification of areas that need to be improved and the adjustments to be made to a mixture’s parameters to meet the specific conditions of a given project. Previous Polish experiments concerning road construction prove that incorrectly defined requirements for a cold recycling mixture may result in the production of an excessively stiff mixture, which will lead to transverse cracking of the finished road surface. Field observations, laboratory tests, and analyses of the causes of fractures led to some modifications of the requirements. This change aimed to create a trend toward designing a cold recycling mixture that would be more elastic. Under laboratory conditions, MCE mixtures designed in line with the new requirements showed lower stiffness, but it is only through further inspections of the road sections currently under construction that we will be able to verify whether the achieved decrease in the mixture’s stiffness is sufficient for eliminating the risk of reflecting cracking. The tests performed in this study showed that more care is needed when designing the composition of a cold recycling MCE mixture in order to obtain the required parameters with the lowest possible content of cement. This is achievable if the designed mixture has the optimum composition (reclaimed material and new aggregate).

To summarize, this interdisciplinary analysis of MCE technology emphasizes the potential of this technology as well as the complexity of problems related to its practical application. It has been demonstrated that providing adequate education and training for road engineers and technicians can help overcome technical and operational barriers, which, at the moment, limit the wider use of this technology. The development of norms and standards better suited to the realities of road construction and use could be of key importance to take full advantage of the benefits offered by MCE mixtures.

## Figures and Tables

**Table 1:** Composition of the MCE mixture used.

No	Material	Share in the MCE Mixture [%]
1	Reclaimed asphalt pavement	64.6
2	Mixture 0/31.5	14.5
3	Medium sand	14.5
4	CEMII B-M (V-LL) 32.5 R	3.0
5	Asphalt cation emulsion C60 B10 ZM/R	3.5

**Table 2:** MCE mixture design—required parameters.

No	Properties	Research Standard	Result	Required	
				Min	Max
1	Content of binder in reclaimed asphalt pavement	PN-EN 12697-1 [56]	5.5	-	-
2	Bulk density of the mineral-cement mixture’s skeleton (method II), g/cm^3^	PN-EN 13286-2 [57]	2.194	-	-
3	Optimum moisture content of the mineral-cement mixture (method II), %	PN-EN 13286-2	4.7	-	-
4	Optimum moisture content of the MCE mixture inclusive of the water contained in emulsion, %	-	3.3	-	-
5	Bituminous binder content (old), %	PN-EN 12697-1	3.5	-	-
6	Bituminous binder content including the asphalt precipitated from emulsion, %	PN-EN 12697-1	5.5	-	-
7	Cement content, %	-	3.0	1.0	4.0
8	Emulsion content, %	-	3.5	2.0	6.0
9	Intermediate tensile strength ITS after 7 days (+5 °C), MPa	PN-EN 12697-23 [58]	0.8	0.5	1.0
10	Intermediate tensile strength ITS after 28 days (+5 °C), MPA	PN-EN 12697-23	1.1	0.7	1.6
11	Water resistance after 28 days (+5 °C), %	PN-EN 12697-23	83.9	80.0	-
12	Modulus of stiffness IT-CY after 28 days (+5 °C), %	PE-EN 12697-26 annex C [59]	5873.0	2000.0	7000.0
13	Density, g/cm^3^	PE-EN 12697-5 [60]	2.521	-	-
14	Bulk density, g/cm^3^	PN-EN 12697-6 [54]	2.145	-	-
15	Content of free spaces, %	PE-EN 12697-8 [61]	14.9	8.0	15.0

**Table 3:** Summary of main test results for the MCE mixture (average ± range).

No	Parameter	Unit	Mean Value	Range		Specification Limit
				Min	Max	
1	ITS (7 days)	MPa	0.81	0.72	0.88	≥0.5
2	ITS (28 days)	MPa	1.09	0.95	1.20	≥0.7
3	Stiffness modulus IT-CY (28 days)	MPa	5873	5100	6250	2000–7000
4	Bulk density	g/cm^3^	2.145	2.12	2.16	-
5	Air void content	%	14.9	13.8	15.2	8–15

## Data Availability

The original contributions presented in this study are included in the article. Further inquiries can be directed to the corresponding author.

## References

[1] Szafranko E., Harasymiuk J., Jurczak M. (2024). Circular economy in construction as an element of the green building philosophy. Proc. Int. Multidiscip. Sci. Geoconf..

[2] Szafranko E. (2025). Circular economy implemented on the construction site. Constr. Optim. Energy Potential/Bud. O Zoptymalizowanym Potencjale Energetycznym.

[3] Dołżycki B., Jaskuła P. (2019). Review and evaluation of cold recycling with bitumen emulsion and cement for rehabilitation of old pavements. J. Traffic Transp. Eng..

[4] Chomicz-Kowalska A., Stępień J. (2016). Cost and eco-effective cold in-place recycled mixtures with foamed bitumen during the reconstruction of a road section under variable load bearing capacity of the subgrade. Procedia Eng..

[5] Firlej S., Kukiełka J. (2019). Load bearing capacity of a nationalroad with base course made of mineral-cement-emulsion mixture after 12 years of exploitation. Roads Bridges—Drog. I Mosty.

[6] Pasetto M., Baldo N. (2011). Cold recycled mixes with cement and bitumen emulsion for sustainable pavements: Mechanical and environmental assessment. Mater. Struct..

[7] Skotnicki Ł., Kuźniewski J., Szydło A. (2021). Research on the properties of mineral–cement emulsion mixtures using recycled road pavement materials. Materials.

[8] Mazurek G., Iwanski M. (2019). Optimisation of the innovative hydraulic binder composition for its versatile use in recycled road base layer. IOP Conf. Ser. Mater. Sci. Eng..

[9] Yang W., Ouyang J., Meng Y., Han B., Sha Y. (2021). Effect of curing and compaction on volumetric and mechanical properties of cold-recycled mixture with asphalt emulsion under different cement contents. Constr. Build. Mater..

[10] Miljković M., Poulikakos L., Piemontese F., Shakoorioskooie M., Lura P. (2019). Mechanical behaviour of bitumen emulsion-cement composites across the structural transition of the co-binder system. Constr. Build. Mater..

[11] Chen T., Luan Y., Ma T., Zhu J., Huang X., Ma S. (2020). Mechanical and microstructural characteristics of different interfaces in cold recycled mixture containing cement and asphalt emulsion. J. Clean. Prod..

[12] Cui K., Chang J. (2022). Hydration, reinforcing mechanism, and macro performance of multi-layer graphene modified cement composites. J. Build. Eng..

[13] Lu D., Jiang X., Zhifei T., Binbin Y., Zhen L., Jing Z. (2023). Enhancing sustainability in pavement Engineering: A-state-of-the-art review of cement asphalt emulsion mixtures. Clean. Mater..

[14] Jain S., Singh B. (2021). Cold mix asphalt: An overview. J. Clean. Prod..

[15] Lin J., Hong J., Xiao Y. (2017). Dynamic characteristics of 100% cold recycled asphalt mixture using asphalt emulsion and cement. J. Clean. Prod..

[16] Su N., Xiao F., Wang J., Amirkhanian S. (2017). Characterizations of base and subbase layers for Mechanistic-Empirical Pavement Design. Constr. Build. Mater..

[17] Iowa State University Institute for Transportation (2020). Pavement Subbase Design and Construction.

[18] Liu L., Li Z., Cai G., Liu X., Yan S. (2020). Humidity field characteristics in road embankment constructed with recycled construction wastes. J. Clean. Prod..

[19] Asphalt Emulsion Manufacturers Association, Asphalt Institute, Federal Highway Administration (2019). Basic Asphalt Emulsion Manual.

[20] Asphalt Recycling & Reclaiming Association (ARRA) (2015). Basic Asphalt Recycling Manual.

[21] Tao M.A., Lu Y.-C., He L., Huang X.-M., Wang S.-Q., Wang N., Yuan M.A. (2023). Review on cold recycling technology development of emulsified asphalt and foamed asphalt. J. Transp. Eng..

[22] Zhao H., Su J., Ma S., Su C., Wang X., Li Z., Wei J., Cui S. (2022). Study on cold recycled asphalt mixtures with emulsified/foamed asphalt in the laboratory and on site. Coatings.

[23] Jin D., Ge D., Chen S., Che T., Liu H., Malburg L., You Z. (2021). Cold In-Place Recycling Asphalt Mixtures: Laboratory Performance and Preliminary M-E Design Analysis. Materials.

[24] Chen Z., Yi J., Zhao H., Luan H., Xu M., Zhang L. (2020). Improved design method of emulsified asphalt cold recycled mixture. Front. Mater..

[25] Yang Y., Yang Y., Qian B. (2019). Performance and microstructure of cold recycled mixes using asphalt emulsion with different contents of cement. Materials.

[26] Gan Y., Zhang X., Jiang Z., Lu D., Xu N., Han L., Han X. (2022). Cementitious fillers in cement asphalt emulsion mixtures: Long-term performance and microstructure. Arab. J. Sci. Eng..

[27] Apeagyei A.K., Diefenderfer B.K. (2013). Evaluation of cold in-place and cold central-plant recycling methods using laboratory testing of field-cored specimens. J. Mater. Civ. Eng..

[28] National Cooperative Highway Research Program (2020). Proposed Practice for Performance-Related Specifications for Cold Recycled Pavements Using Asphalt Emulsions and Foamed Asphalt (Report 952).

[29] Liu L., Li Z., Cai G., Congress S.S., Liu X., Dai B. (2020). Evaluating the influence of moisture on settling velocity of road embankment constructed with recycled construction wastes. Constr. Build. Mater..

[30] Norambuena-Contreras J., Quilodran J., Gonzalez-Torre I., Chavez M., Borinaga-Treviño R. (2018). Electrical and thermal characterisation of cement-based mortars containing recycled metallic waste. J. Clean. Prod..

[31] Montepara A., Tebaldi G., Marradi A., Betti G. (2012). Effect on pavement performance of a subbase layer composed by natural aggregate and RAP. Procedia-Soc. Behav. Sci..

[32] Gómez-Meijide B., Pérez I. (2014). Effects of the use of construction and demolition waste aggregates in cold asphalt mixtures. Constr. Build. Mater..

[33] Pasandín A.R., Pérez I. (2017). Fatigue performance of bituminous mixtures made with recycled concrete aggregates and waste tire rubber. Constr. Build. Mater..

[34] Kolo S.S., Shehu M., Abdulrahman H.S., Adamu H.N., Eso O.S., Eya S.A. (2024). Mechanical properties of cold mixed asphalt. Eng. Today.

[35] Terrones Saeta J.M., Iglesias Godino F.J., Corpas Iglesias F.A., Martínez García C. (2020). Study of the incorporation of ladle furnace slag in the manufacture of cold in place recycling with bitumen emulsion. Materials.

[36] Stępień J., Maciejewski K. (2022). Using reclaimed cement concrete in pavement base mixes with foamed bitumen produced in cold recycling technology. Materials.

[37] Gómez Meijide B., Pérez I. (2014). A proposed methodology for the global study of the mechanical properties of cold asphalt mixtures. Mater. Des..

[38] Li Y., Guo M., Tan Y., Wang L. (2019). Effects of cement and emulsified asphalt on properties of mastics and 100% cold recycled asphalt mixtures. Materials.

[39] Pi Y., Huang Z., Pi Y., Li G., Li Y. (2019). Composition design and performance evaluation of emulsified asphalt cold recycled mixtures. Materials.

[40] Jiang Y., Lin H., Han Z., Deng C. (2019). Fatigue properties of cold recycled emulsified asphalt mixtures fabricated by different compaction methods. Sustainability.

[41] Bessa I.S., Almeida L.R., Vasconcelos K.L., Bernucci L.L. (2016). Design of cold recycled mixes with asphalt emulsion and portland cement. Can. J. Civ. Eng..

[42] Oruc S., Celik F., Akpınar M.V. (2007). Effect of cement on emulsified asphalt mixtures. J. Mater. Eng. Perform..

[43] Almusawi A., Jaleel M.M., Shoman S., Lupanov A.P. (2024). Enhancing waste asphalt durability through cold recycling and additive integration. Funct. Compos. Mater..

[44] Mignini C., Cardone F., Graziani A. (2018). Experimental study of bitumen emulsion–cement mortars: Mechanical behaviour and relation to mixtures. Mater. Struct..

[45] Kukiełka J., Gałan K. (2023). The influence of grain size composition on the stiffness modulus of mineral-cement-emulsion mixtures (MCEM) with the rubber powder addition. Roads Bridges-Drog. I Mosty.

[46] Buczyński P., Krasowski J. (2024). Optimisation and composition of the recycled cold mix with a high content of waste materials. Sustainability.

[47] Pi Y., Li Y., Pi Y., Huang Z., Li Z. (2019). Strength and micro-mechanism analysis of cement-emulsified asphalt cold recycled mixture. Materials.

[48] Du S. (2015). Performance characteristic of cold recycled mixture with asphalt emulsion and chemical additives. Adv. Mater. Sci. Eng..

[49] IBEF (2025). Recycling with Bitumen Emulsions: Technical, Economic and Environmental Benefits. *Emulsions—Techniques*, IBEF. https://www.ibef.net/en/emulsions-2/techniques/recycling-with-bitumen-emulsions/.

[50] Wang D., Yao H., Yue J., Hu S., Liu J., Xu M., Chen S. (2021). Compaction characteristics of cold recycled mixtures with asphalt emulsion and their influencing factors. Front. Mater..

[51] Recasens R.M., Pérez Jiménez F.E., Aguilar S.C. (2000). Mixed recycling with emulsion and cement of asphalt pavements. Design procedure and improvements achieved. Mater. Struct..

[52] (2017). Asphalt Mineral-Asphalt Mixtures—Test Methods for Samples Taken from the Surface.

[53] (2012). Annex C—Appendix C—Test Methods.

[54] (2020). Test Methods—Part 6: Determination of Bulk Density of Mixture Samples.

[55] (2005). Methods for Testing the Stability of Asphalt Mixtures (Including BBTM Mixtures).

[56] (2020). Asphalt Mixtures. Test Methods. Part 1: Soluble Binder Content.

[57] (2010). Methods for Determining Density and Moisture.

[58] (2017). Tests of Mineral-Asphalt Mixtures, Methodology for Determining Their Indirect Tensile Strength.

[59] (2017). Annex C—Methods for Testing the Stiffness of Asphalt Mixtures.

[60] (2010). Asphalt Mixtures. Test Methods. Binder Content Determination.

[61] (2003). Asphalt Mixtures—Test Methods—Determination of the Abrasion Angle Constant.

[62] (2012). Grain Shape Testing.

[63] (2001). Aggregate Testing Methods, Determination of the Abrasion Angle Constant.

